# Photochemical disruption of endocytic vesicles before delivery of drugs: a new strategy for cancer therapy

**DOI:** 10.1038/sj.bjc.6600138

**Published:** 2002-02-12

**Authors:** L Prasmickaite, A Høgset, P K Selbo, BØ Engesæter, M Hellum, K Berg

**Affiliations:** Department of Biophysics, Institute for Cancer Research, The Norwegian Radium Hospital, Montebello, N-0310 Oslo, Norway; Department of Tumor Biology, Institute for Cancer Research, The Norwegian Radium Hospital, Montebello, N-0310 Oslo, Norway; PCI Biotech AS, Hoffsvn. 48, N-0377 Oslo, Norway

**Keywords:** photochemical, endocytic vesicles, drug delivery, cancer therapy

## Abstract

The development of methods for specific delivery of drugs is an important issue for many cancer therapy approaches. Most of macromolecular drugs are taken into the cell through endocytosis and, being unable to escape from endocytic vesicles, eventually are degraded there, which hinders their therapeutic usefulness. We have developed a method, called photochemical internalization, based on light-induced photochemical reactions, disrupting endocytic vesicles specifically within illuminated sites e.g. tumours. Here we present a new drug delivery concept based on photochemical internalization-principle – photochemical disruption of endocytic vesicles before delivery of macromolecules, leading to an instant endosomal release instead of detrimental stay of the molecules in endocytic vesicles. Previously we have shown that illumination applied after the treatment with macromolecules substantially improved their biological effect both *in vitro* and *in vivo*. Here we demonstrate that exposure to light before delivery of protein toxin gelonin improves gelonin effect *in vitro* much more than light after. However, *in vitro* transfection with reporter genes delivered by non-viral and adenoviral vectors is increased more than 10- and six-fold, respectively, by both photochemical internalization strategies. The possible cellular mechanisms involved, and the potential of this new method for practical application of photochemical internalization concept in cancer therapy are discussed.

*British Journal of Cancer* (2002) **86**, 652–657. DOI: 10.1038/sj/bjc/6600138 www.bjcancer.com

© 2002 Cancer Research UK

## 

Despite exciting progress in the development of new anticancer agents, the development of appropriate delivery strategies, allowing a therapeutic molecule to reach the target and express a therapeutic function, remains to be one of the most important issues for cancer therapy. Most of anticancer agents (e.g. protein- or nucleic acid-based drugs, some watersoluble chemotherapeutics) are membrane-impermeable big molecules, usually taken into the target cell through the endocytic pathway. Moreover, to target tumour cells, therapeutic molecules are often coupled to different ligands, binding to specific receptors on tumour cells, which also leads to receptor-mediated endocytosis (Dubowchik and Walker, 1999). However, macromolecules usually do not have means for escaping from endocytic vesicles and consequently, degradation inside these vesicles in many cases hinders their therapeutic application (Lloyd, 2000). Thus, it is known that type I ribosome-inactivating protein toxins could have a great potential as anticancer agents if released from the vesicles (Barbieri *et al*, 1993). Also, inefficient endosomal release of therapeutic nucleic acids, especially if delivered by non-viral vectors, is an important obstacle for cancer gene therapy (Zabner *et al*, 1995; Luo and Saltzman, 2000). Therefore, the development of endosome-disruptive strategies to liberate therapeutic macromolecules is of great importance for the exploitation of full potential of anticancer drugs.

Recently we have developed a new technology named photochemical internalization (PCI) to improve the cytosolic delivery of various macromolecules that are taken into the cell via endocytosis (Berg *et al*, 1999). The method is based on the use of photosensitizing compounds, such as aluminium phthalocyanine (AlPcS_2a_) that localize in endocytic vesicles and upon light exposure induce photochemical damage to the vesicular membranes, releasing intact macromolecules into the cytosol (Berg *et al*, 1999). PCI, as a light-dependent treatment, is an attractive approach for targeted drug delivery, since in principal only areas exposed to light (e.g. tumours) will be subjected to the drug effects.

We have reported earlier that the PCI principle can be utilized *in vitro* for transfer of various macromolecules. Thus, the toxicity of proteins, such as the type I ribosome-inactivating toxin gelonin and a tumour-targeted immunotoxin, was considerably increased by photochemical treatment (Berg *et al*, 1999; Selbo *et al*, 2000). Likewise, the efficiency of transfection with a reporter transgene was improved 10–20-fold by light (Høgset *et al*, 2000; Prasmickaite *et al*, 2000). We have also demonstrated the potential of the PCI technology *in vivo* in an animal model, where the cytotoxic effect of gelonin, which was completely inactive when used alone, was increased tremendously by photochemical treatment, resulting in a complete regression of subcutaneous tumours in mice (Selbo *et al*, 2001).

Originally the PCI method was developed to liberate macromolecules, which had already been endocytosed and trapped in endocytic vesicles, i.e. it was based on illumination applied after the treatment with macromolecules (‘light after’ strategy). Although the technology generally works well in this setting, there are several potential disadvantages: (i) the possible enzymatic degradation of the macromolecules in endocytic vesicles before the liberation; (ii) the possible induction of photochemical damage to the macromolecules located close to the photosensitizer at the moment of illumination; (iii) the inconvenience of a sequential treatment in a therapeutic situation. Therefore, the aim of this study was to investigate the possibility of disrupting endocytic vesicles in advance, i.e. before the macromolecules are taken into the cell (‘light before’ strategy). In this report we show that this novel PCI approach can increase the biological effect of both protein toxins and transfecting nucleic acids more or at least as efficiently as our previously described strategy. The implication of the findings for the development of light-directed drug delivery systems for cancer therapy will be discussed.

## MATERIALS AND METHODS

### Reagents and cell culture

A plasmid pEGFP-N1 (encoding Enhanced Green Fluorescent Protein (EGFP)) was purchased from Clontech Laboratories, Inc. (Palo Alto, CA, USA), AlPcS_2a_ was from Porphyrin Products (Logan, UT, USA). Gelonin, poly-L-lysine hydrobromide (MW 20700) and FITC-dextran (MW 4400) were from Sigma (St. Louis, MO, USA). Fluorescein di-β-D-galactopyranoside was from Molecular Probes (Eugene, OR, USA). Adenovirus AdHCMV-lacZ was kindly provided by Dr FL Graham, McMaster University, Ontario, Canada.

The human malignant melanoma cell line THX was established from a tumour tissue obtained from a patient treated for metastatic malignant melanoma at the Norwegian Radium Hospital. The cells were cultured in RPMI-1640 medium supplemented with 10% foetal calf serum, 100 units ml^−1^ penicillin, 100 μg ml^−1^ streptomycin and 2 mM glutamine (all from Bio Whittaker, Walkersville, MD, USA) at 37°C in 5% CO_2_ atmosphere.

### Treatment with different macromolecules

For the treatment with gelonin 25×10^3^ cells per well were seeded out into 24-well plates (Nunc, Denmark). For the transfection with pEGFP-N1/polylysine and transduction with AdHCMV-lacZ 50×10^3^ THX cells per well were seeded out into 6-well plates. The next day 20 μg ml^−1^ AlPcS_2a_ were added, and the cells were incubated for 18 h at 37°C. All the procedures after AlPcS_2a_ addition were carried on in subdued light. For the ‘light before’ strategy AlPcS_2a_ was removed, the cells were washed three times and incubated in AlPcS_2a_-free medium for 4 h. Then the cells were exposed to light (see below) before the treatment with selected macromolecules: either with gelonin (1 μg ml^−1^ for 18 h), or with the pEGFP-N1/polylysine complex (5 μg ml^−1^ pEGFP-N1, for different time depending on the experiment), or with AdHCMV-lacZ (at a multiplicity of infection (MOI) 1 for 30 min). For the ‘light after’ strategy the cells were first treated with the selected macromolecules at the same concentrations and for the same time as indicated above, washed, and after addition of fresh culture medium exposed to light. Non-illuminated cells were treated in a similar way except for illumination.

The treated cells were washed once with culture medium, and after addition of fresh medium incubated at 37°C before further analysis. Inhibition of protein synthesis was assayed by [^3^H]leucine incorporation into proteins 24 h after light exposure as previously described (Llorente *et al*, 1998). EGFP and β-galactosidase expression was analyzed by flow cytometry (see below) 2 days after illumination.

Illumination was performed from a bench of four light tubes (Philips TLD 18W/79) and a long pass filter with a cut off at 550–600 nm. The light intensity reaching the cells was 13.5 W m^−2^. Light dose used for transgene delivery induces approximately 50% toxicity (i.e. D_50_).

pEGFP-N1/polylysine complex (charge ratio 1.7) was prepared by gently mixing plasmid and polylysine solutions prepared separately: 5 μg pEGFP-N1 plasmid was diluted in 75 μl water and 5.3 μg polylysine diluted in 75 μl water. The solutions were mixed and incubated for 30 min at room temperature, diluted with culture medium to 1 ml and added to the cells.

### Flow cytometry analysis

For the detection of EGFP, the cells were resuspended in 400 μl of culture medium and analyzed by a FACS-Calibur flow cytometer (Becton Dickinson, San Jose, CA, USA). For each sample 10 000 events were collected. EGFP was measured through a 510–530 nm filter after excitation with an argon laser (15 mW, 488 nm). Dead cells were discriminated from single viable cells by gating on forward scattering *vs* side scattering. The data were analyzed with CELLQuest Software (Becton Dickinson).

The expression of β-galactosidase was measured as previously described (Nolan *et al*, 1988). Briefly, the cells were resuspended in 25 μl of culture medium and incubated for 5 min at 37°C. 25 μl of 2 mM fluorescein di-β-D-galactopyranoside were added and incubated for 1 min at 37°C. The suspension was diluted by adding 450 μl ice cold culture medium, and the samples were maintained on ice for 30–60 min before analysis by flow cytometry using the same settings as described above.

### Fluorescence microscopy

THX cells were seeded out into Falcon 3001 dishes (25×10^3^ cells per dish) and the next day treated with 20 μg ml^−1^ AlPcS_2a_ for 18 h, washed and incubated in AlPcS_2a_-free medium for 4 h. Then the cells were exposed to light for 4 min before a 3 h incubation with 5 mg ml^−1^ FITC-dextran. Non-illuminated cells were treated in a similar way except for illumination. The intracellular localization of FITC-dextran in unfixed cells was observed with a Zeiss Axioplan fluorescence microscope (Oberkochen, Germany) using an objective with 63×magnification, a 450–490 nm band pass excitation filter and a 510–540 band pass emission filter. Fluorescence micrographs were recorded by means of a cooled charge-coupled device (CCD) camera (Photometrics Inc., Tucson, AZ, USA).

## RESULTS

### Effect of photochemical treatment on the intracellular localization of an endocytic marker

**Figure 1:**
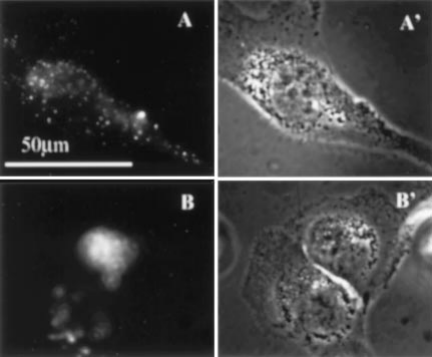
Effect of photochemical treatment on intracellular localization of FITC-dextran. (**A,B**) Fluorescence micrographs. THX cells were incubated with 20 μg ml^−1^ AlPcS_2a_ for 18 h followed by a 4 h incubation in AlPcS_2a_-free medium. Then the cells were either kept in the darkness (**A**) or exposed to light for 4 min (**B**) before a 3 h incubation with 5 mg ml^−1^ FITC-dextran. (**A**′**,B**′) Phase contrast showing the morphology of the corresponding cells.

First we studied by fluorescence microscopy, whether photochemical treatment applied before the incubation with an endocytic marker (FITC-dextran) affects the intracellular localization of the marker. As can be seen in Figure 1A

, cells that were treated with AlPcS_2a_ and FITC-dextran in the absence of light show granular FITC fluorescence, indicating, as expected, localization of FITC-dextran in endocytic vesicles. In contrast, exposure to light before the incubation with FITC-dextran leads to diffuse FITC fluorescence (Figure 1B), suggesting that a substantial fraction of FITC-dextran entered the cytosol instead of accumulating in endocytic vesicles. However, when AlPcS_2a_-treated cells were exposed to light followed by incubation with FITC-dextran at 4°C, when endocytic uptake is inhibited, no intracellular FITC fluorescence was detected (data not shown), indicating that: (i) endocytosis is important for the uptake of FITC-dextran into photochemically treated cells; (ii) photochemical treatment does not permeabilize cellular membrane so that FITC-dextran could passively diffuse into the cell cytosol.

### Effect of photochemical treatment on the cytotoxic effect of gelonin

**Figure 2:**
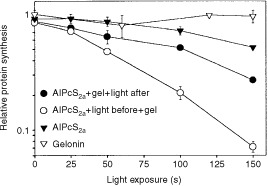
Effect of photochemical treatment on gelonin-induced inhibition of protein synthesis. For the ‘light before’ strategy the cells were first incubated with 20 μg ml^−1^ AlPcS_2a_ for 18 h, then for another 4 h in AlPcS_2a_-free medium before exposure to light as indicated in the figure. After illumination 1 μg ml^−1^ gelonin (gel) was added, and the cells were incubated for 18 h. For the ‘light after’ strategy the cells were co-incubated with 20 μg ml^−1^ AlPcS_2a_ and 1 μg ml^−1^ gelonin for 18 h before light exposure. The control cells were treated only with 1 μg ml^−1^ gelonin for 18 h and exposed to light, or only with 20 μg ml^−1^ AlPcS_2a_ for 18 h, chased 4 h in AlPcS_2a_-free medium and exposed to light. [^3^H]leucine incorporation into proteins was measured the day after the light-treatment. Data presented are the mean relative to non-illuminated cells. Data points represent mean±s.e. of triplicates.

The protein toxin gelonin is taken into the cell via endocytosis, but is trapped in endocytic vesicles and finally degraded, therefore it is relatively non-toxic to intact cells. However, in cell-free systems gelonin is a very potent inhibitor of protein synthesis, indicating that the inability to escape from endocytic vesicles is the major cause for the low toxicity to intact cells (Stirpe *et al*, 1980). As can be seen in Figure 2

, gelonin had no effect on protein synthesis when applied in the absence of photochemical treatment. However, exposure of AlPcS_2a_-treated cells to light before the incubation with gelonin considerably increased the cytotoxicity of gelonin. Comparison of the ‘light before’ and the ‘light after’ approaches in THX cells shows that gelonin exhibited much stronger toxicity when light was applied before gelonin-treatment as compared to light after (Figure 2).

### Effect of photochemical treatment on gene transfection

**Figure 3:**
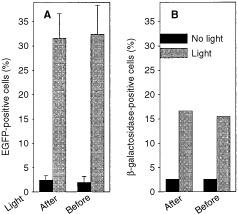
(**A**) Effect of photochemical treatment on transfection with pEGFP-N1/polylysine complex. For the ‘light before’ strategy AlPcS_2a_-pretreated THX cells were incubated in AlPcS_2a_-free medium for 4 h before light exposure for 3 min (corresponding approximate D_50_ dose). Following illumination pEGFP-N1/polylysine (5 μg ml^−1^ plasmid) was added and the cells were incubated for 4 h. For the ‘light after’ strategy AlPcS_2a_-treated cells were transferred into AlPcS_2a_-free medium and incubated with the pEGFP-N1/polylysine complex for 4 h, transferred into complex-free medium and exposed to light. After 2 days the cells were analyzed for EGFP expression. Each bar represents the mean±s.e. of 5–7 experiments. (**B**) Transduction with AdHCMV-lacZ. For the ‘light before’ strategy AlPcS_2a_-pretreated THX cells were incubated for another 4 h in AlPcS_2a_-free medium before light exposure for 3 min (corresponding approximate D_50_ dose). Following illumination the cells were infected with AdHCMV-lacZ (at MOI 1) for 30 min, 2 ml of medium was added and after 2 days the cells were analyzed for β-galactosidase expression. For the ‘light after’ strategy AlPcS_2a_-treated cells were incubated in AlPcS_2a_-free medium for 3 h before a 30 min infection with AdHCMV-lacZ. After addition of 2 ml of culture medium the cells were incubated for another 30 min before illumination. Two days later the cells were analyzed for β-galactosidase expression.

The effect of photochemical treatment on delivery of transgenes was studied by measuring the efficiency of transfection with a plasmid, containing a reporter EGFP-gene driven by the cytomegalovirus promoter. The cationic polypeptide poly-L-lysine was used for DNA complexation, and the transfection efficiency was evaluated measuring EGFP expression. As can be seen in Figure 3A

, light exposure increased the amount of EGFP-positive cells by >10-fold as compared to non-illuminated cells, irrespectively of whether light was applied before or after the DNA complex.

Photochemical treatment employing either ‘light before’ or ‘light after’ strategy also stimulated transfection mediated by the targeted transfection agent transferrin-poly-L-lysine (data not shown).

### Effect of photochemical treatment on adenovirus-mediated gene transduction

In order to study the effect of illumination before gene transduction with viruses (the most used vectors in cancer gene therapy clinical trials), we used the adenovirus AdHCMV-lacZ, containing a β-galactosidase reporter gene controlled by the cytomegalovirus promoter. The cells were pre-incubated with AlPcS_2a_ followed by light exposure before or after a 30 min incubation with AdHCMV-lacZ. As can be seen in Figure 3B, the percentage of β-galactosidase-expressing cells was increased approximately six-fold by both light-treatments, as compared to control cells that were infected with the adenovirus, but not exposed to light. Higher virus doses (i.e. higher MOI) gave higher percentage of transducted cells in both non-illuminated and photochemically treated cell populations (data not shown), however, in general, fold-increase achieved by photochemical treatment is the highest for low virus doses.

### Effect of illumination time point

**Figure 4:**
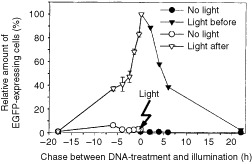
Effect of illumination time point on the efficiency of light-induced transfection with pEGFP/polylysine. For the ‘light before’ strategy AlPcS_2a_-pretreated THX cells were transferred into AlPcS_2a_-free medium and incubated for 4 h before light exposure for 3 min. Following illumination the cells were either immediately incubated with pEGFP/polylysine complex (5 μg ml^−1^ plasmid) for 30 min or chased in culture medium for different periods (as indicated in the scheme) before a 30 min incubation with pEGFP/polylysine. For the ‘light after’ strategy AlPcS_2a_-treated cells were transferred into AlPcS_2a_-free medium, incubated with pEGFP/polylysine complex for 30 min and either immediately exposed to light or chased for different periods in complex-free medium before illumination. After treatments the cells were transferred into fresh culture medium and incubated for 2 days before analysis of EGFP expression. The relative EGFP expression was calculated taking as 100% the percentage of EGFP-expressing cells, when the pEGFP/polylysine-treatment and the light-treatment followed each other without any chase (*t*=0). Data points represent mean±s.e. of three experiments.

For the potential use of PCI as a method for light-induced delivery *in vivo*, it was important to define the ‘time window’ for achieving the maximal effect. Thus, THX cells were given a 30 min pulse with the DNA/polylysine complex and were illuminated at different time points before or after the DNA pulse. As can be seen in Figure 4

, transfection using the ‘light before’ strategy was most efficient when light was given right before the DNA complex. A 5 h delay reduced the efficiency of transfection by approximately 50%, suggesting that the transfection-enhancing effects induced by light last for up to at least 5 h, albeit decreasing with time. For comparison, using the ‘light after’ strategy, the highest amount of EGFP-positive cells was obtained when light was applied immediately after the DNA complex, indicating that both the DNA-treatment and the light-treatment should be strongly co-ordinated in time, optimally following each other without any delay.

## DISCUSSION

**Figure 5:**
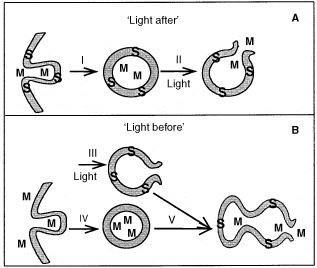
Photochemical internalization: ‘light after’ and ‘light before’ induced cytosolic delivery of endocytosed macromolecules. (**A**) ‘Light after’: both the photosensitizer (S) and the macromolecule (M) are endocytosed and localize in the same endocytic vesicles (I). Light exposure induces photochemical reactions, leading to the disruption of vesicular membranes and resulting in cytosolic release of the macromolecules (II). (**B**) ‘Light before’: light-induced disruption of vesicular membranes containing S (III) before M is endocytosed and localize in intact endocytic vesicles (IV). Fusion between intact M-containing and photochemically disrupted S-containing vesicles leads to the cytosolic release of the macromolecules (V).

In this study we present a new drug delivery principle – photochemical disruption of endocytic vesicles in specific regions of the body, for improving internalization of later applied macromolecular drugs. This photochemical method offers for cancer therapy a way for obtaining drug effects specifically in tumour cells. The technology can be used with various macromolecules that are taken into the cell by endocytosis, and whose therapeutic activity is normally hampered by the inability to escape from endocytic vesicles. Unlike our earlier described method (Berg *et al*, 1999; Høgset *et al*, 2000; Selbo *et al*, 2000, 2001), where illumination was performed after the treatment with macromolecules ( Figure 5A

), and unlike most other methods for endosomal liberation, the strategy presented in this paper suggests permeabilization of endocytic vesicles before delivery of macromolecules, avoiding a prolonged and possibly detrimental stay of the molecules in endocytic vesicles. In effect this leads to a very rapid relocalization of the endocytosed macromolecules into the cytosol, and consequently improves their biological effect. In this study we demonstrate that ‘light before’ PCI can substantially enhance the cytotoxicity of protein toxins as well as efficiency of gene transfection mediated by both non-viral and viral vectors.

There are several reasons why illumination before drug delivery should be advantageous as compared to previously discussed ‘light after’ strategy. Firstly, in most clinical situations it would be practically advantageous if the light-treatment and the administration of the drugs could be performed as simultaneously as possible. For example, employing the technology in combination with surgery, as an adjuvant treatment of residual disease after surgical removal of tumours, it would be a great advantage to apply both the light-treatment and the therapeutic macromolecule at the end of surgical procedures and immediately following each other. In this case the ‘light before’ approach would be clearly advantageous to the ‘light after’ strategy, where one would have to wait several hours to ensure maximal uptake of the drug. Secondly, the ‘light before’ strategy could be more optimal for delivery of intact macromolecules. Photochemical treatment can damage various biomolecules (Jori and Spikes, 1984). Therefore photochemical damage to the entrapped therapeutic molecules can not be ruled out when light is applied after the molecules, and this could decrease the positive effects of PCI. However, the primary photochemical effects should not directly damage molecules that are delivered into the cell after illumination. Thirdly, it is known that photochemical treatment can at least partially inactivate degradative lysosomal enzymes or impair microtubules involved in transport towards lysosomes (Berg and Moan, 1994, 1997), so that if applied in advance, it could protect endocytosed macromolecules from the possible lysosomal degradation by both these mechanisms.

In this study it has been shown that both the ‘light before’ and the ‘light after’ PCI-strategies improve the final biological effect of delivered macromolecules, however which approach leads to the better result seems to depend on the macromolecules to be delivered. Thus, in the case of gelonin it seems to be more beneficial to apply ‘light before’ gelonin (Figure 2). However, in the case of transgene, light-induced transfection mediated by both polylysine and adenovirus was similar regardless of whether illumination was performed before or after administration of the transgene (Figure 3). The difference in response between these two classes of molecules might be due to their different sensitivity, either to enzymatic degradation in endocytic vesicles, or to the photochemically induced damage. If gelonin is more sensitive to these damaging effects than the transgene (that might be protected by the carrier molecules: synthetic vectors or viruses), then the “‘light before’ approach might be especially beneficial due to the reasons discussed above. It should be mentioned that gelonin and reporter transgenes were used as examples to demonstrate the versatility and efficacy of the technology. However, the method is far from being optimized, and by use of other macromolecules under optimal conditions even better effects could be expected.

It has already been shown the efficiency of ‘light after’ PCI strategy *in vivo* in combination with gelonin (Selbo *et al*, 2001). The results described in the present work show that, in addition to the above mentioned practical advantages, the ‘light before’ strategy with gelonin *in vitro* works substantially more efficiently, making the presented new PCI concept worth further investigation. The exact cellular mechanism behind ‘light before’-based delivery is not known. One obvious possibility – the direct entry into the cytosol through the plasma membrane of photochemically treated cells – does not seem very likely, since the results argue against photochemical permeabilization of the plasma membrane and strongly indicate the involvement of endocytic transport.

Another alternative is endosomal release mediated by passive diffusion. As discussed above, photochemical treatment, if applied in advance, could protect endocytosed macromolecules from lysosomal degradation, so that functional macromolecules could release from intact endocytic vesicles via slow diffusion. However, the very rapid relocalization of FITC-dextran (Figure 1B) strongly suggests the existence of other mechanisms than slow passive diffusion responsible for PCI effect.

Thus, the likely mechanism behind ‘light before’ effects could be a fusion between photochemically ruptured vesicles and intact vesicles carrying the macromolecules, leading to endosomal release of the molecules as described in Figure 5B. More detailed studies of the light effects on transgene delivery revealed that for the ‘light before’ approach the best result was obtained when light was applied right before the DNA complex, while introducing a chase period between illumination and the DNA-treatment reduces the transfection efficiency (Figure 4). Possible reasons for this can be either cellular repair or removal of photochemically disrupted vesicles, so that the DNA is not redirected into the cytosol, but rather stays in intact vesicles. Work is in progress in our laboratory to prove these hypotheses.

The technology presented in this study represents an important addition to the emerging field of physically induced drug delivery methods. There are several factors making the PCI-technology a relatively tumour-specific method: (i) light, activating photosensitizers and inducing photochemical reactions, can be directed precisely to tumours; (ii) photosensitizers accumulate preferentially in tumours as compared to normal tissue (Chan *et al*, 1990; Pass, 1993). It should also be emphasized that there is already significant clinical experience employing photochemical principles, namely in photodynamic therapy (PDT), a quite specific approach used for treatment of different tumour types (Dougherty *et al*, 1998). In addition, the presented technology involves endocytosis and therefore it is very well suited for combination with other tumour-targeting strategies like surface-targeting via ligand-receptor interaction for specific uptake into tumour cells. Although the limited light penetration through the tissues might be a limitation, by the use of fibre optic devices it is possible to reach many sites also within the body, such as the gastrointestinal tract, lungs, brain etc. (Pass, 1993). Another factor, which might limit application of PCI technology is cytotoxicity, inducible by photochemical treatment, that was successfully exploited in PDT of cancer (Pass, 1993; Dougherty *et al*, 1998). In this respect, an attractive approach could be to use photochemically internalized therapeutic molecules (e.g. toxins or suicide genes) as adjuvants for PDT, to destroy cancer cells that were not efficiently killed by PDT. In this way deeper tumour layers, where due to limited light penetration a lower photochemical dose (consequently, lower toxicity) is obtained, could be also affected. However, in general, to balance photochemical dose is a very important task to exploit the full potential that photochemical internalization offers for different therapies.
